# Measuring and mitigating PCR bias in microbiota datasets

**DOI:** 10.1371/journal.pcbi.1009113

**Published:** 2021-07-06

**Authors:** Justin D. Silverman, Rachael J. Bloom, Sharon Jiang, Heather K. Durand, Eric Dallow, Sayan Mukherjee, Lawrence A. David

**Affiliations:** 1 College of Information Science and Technology, Pennsylvania State University, State College, Pennsylvania, United States of America; 2 Institute for Computational and Data Science, Pennsylvania State University, State College, Pennsylvania, United States of America; 3 Department of Medicine, Pennsylvania State University, Hershey, Pennsylvania, United States of America; 4 Center for Genomics and Computational Biology, Duke University, Durham, North Carolina, United States of America; 5 University Program for Genetics and Genomics, Duke University, Durham, North Carolina, United States of America; 6 Department of Molecular Genetics and Microbiology, Duke University, Durham, North Carolina, United States of America; 7 Departments of Statistical Science, Mathematics, Computer Science, Biostatistics & Bioinformatics, Duke University, Durham, North Carolina, United States of America; Helmholtz-Zentrum fur Infektionsforschung GmbH, GERMANY

## Abstract

PCR amplification plays an integral role in the measurement of mixed microbial communities via high-throughput DNA sequencing of the 16S ribosomal RNA (rRNA) gene. Yet PCR is also known to introduce multiple forms of bias in 16S rRNA studies. Here we present a paired modeling and experimental approach to characterize and mitigate PCR NPM-bias (PCR bias from non-primer-mismatch sources) in microbiota surveys. We use experimental data from mock bacterial communities to validate our approach and human gut microbiota samples to characterize PCR NPM-bias under real-world conditions. Our results suggest that PCR NPM-bias can skew estimates of microbial relative abundances by a factor of 4 or more, but that this bias can be mitigated using log-ratio linear models.

## Author summary

High-throughput DNA sequencing is often used to profile the species composition of host-associated microbial communities (microbiota). One important step in DNA sequencing is amplification where DNA from many different bacteria are repeatedly copied using a technique called Polymerase Chain Reaction (PCR). However, PCR is known to introduce multiple forms of bias as DNA from some bacteria are more efficiently copied than others. Here we introduce experimental and computational procedures that allows PCR NPM-bias (PCR bias from non-primer-mismatch sources) to be measured and mitigated in studies of microbial communities.

This is a *PLOS Computational Biology* Methods paper.

## Introduction

Polymerase Chain Reaction (PCR) amplification is an integral experimental step when profiling microbial communities by high-throughput DNA sequencing of the 16S rRNA gene [1]. Yet, bias introduced by differing amplification efficiencies between templates impedes evaluating community structure [2]. This bias has been repeatedly shown to be a substantial source of error for 16S rRNA studies [3–8] as well as in quantitative PCR (qPCR) studies [9, 10], environmental DNA studies [11], metabarcoding studies [12–14], and DNA methylation studies [15]. Mock libraries have been used to demonstrate that over-amplification of specific templates occurs reproducibly, often with preferential amplification of over 3.5 fold [6]. Even single nucleotide mismatches between primer and template have been shown to lead to preferential amplification of up to 10 fold [16]. Despite substantial experimental effort aimed at optimizing multi-template PCR, including limiting the number of PCR cycles [12], optimizing primers [17], and optimizing polymerases [13, 18], PCR bias remains both incompletely understood and a substantial source of error in microbiome studies [18].

PCR bias likely originates from multiple potentially distinct processes. For example, bias due to primer mismatch occurs primarily in the first three cycles of PCR: After three cycles, the primer binding sequence of the original DNA has been replaced by a sequence complementary to the primers themselves [19, 20]. Yet, studies of mid-to-late cycle PCR demonstrate that non-primer-mismatch sources of primer bias (PCR NPM-bias) can also be substantial. Early work on multi-template PCR demonstrated that, between cycles 10 and 35, the composition of a two-template mixture becomes increasingly biased [7]. More recently, studies of environmental DNA showed that community richness decreases by a factor of approximately four between cycles 10 and 15 alone [21]. Yet, even within mid-to-late stage PCR, it is unclear the extent to which introduced biases are consistent between cycles or differ, for example, due to concentration dependent phenomena.

To correct these biases in microbiome studies, methods have been proposed that involve DNA sequencing of mock communities. McLaren et al. [8], recently proposed a mock community based approach that modeled PCR bias as a compositional perturbation (*i.e*., a translation in the log-ratio of relative abundances between any two taxa). The authors then fit their model to sequenced mock communities with known starting composition to infer and correct for PCR bias. However, mock communities require assembling relevant and comprehensive sets of bacterial taxa for a given sample type, and this approach may not be possible for microbes that cannot be cultured and isolated [22]. Moreover, measurement error in the creation of mock communities may confound estimates of PCR bias as both would appear as a translations in log-ratios [8]. There is therefore a need for approaches that measure and mitigate PCR bias in microbiome studies without the use of mock communities.

Rather than developing experimental approaches for correcting PCR bias, a fruitful alternative has involved computational approaches. For metabarcoding, Pawluczyk et al. (2015) suggested that if isolate samples are available, qPCR bias of isolates could be used to predict and correct PCR bias in DNA sequencing studies. For RNA-seq studies, Baumann and Doerge (2012) suggested a Poisson clustering approach to correct PCR bias based on the abundance distribution of reads for each gene depending on the genomic location of the first base in the read. Also for RNA-seq, *alpine* was recently proposed as a means of inferring and correcting PCR bias based on the use of a reference genome against which transcripts can be aligned [5]. For DNA methylation studies, Moskalev et al. develop a calibration curve based on templates with known methylation patterns.

Early work on PCR amplification of multi-template mixtures of bacterial 16S rRNA provides insights for adapting computational corrections to PCR bias in the setting of modern microbiome research. Over 20 years ago, Suzuki and Giovannoni demonstrated that a simple log-ratio linear model explained PCR bias when amplifying a two template 16S rRNA mixture [7]. Their model stated that if the true ratio of the two 16S rRNA genes prior to PCR was given by *a*_1_/*a*_2_, then the ratio of 16S rRNA genes would be *a*_1_/*a*_2_ × (*b*_1_/*b*_2_)^*x*^ after *x* cycles of PCR. That is, each cycle of PCR amplifies each transcript *j*, on average, *b*_*j*_ times where *b*_*j*_ is often less than a perfect doubling (*b*_*j*_ = 2). Still, applying this simple model to microbiome data presents challenges. First, all pairwise ratios must be modeled simultaneously, which requires multivariate extensions of simple pair-wise log-ratios. Second, unlike the measurements of Suzuki and Giovannoni, 16S rRNA sequencing are zero-laden and require modeling approaches appropriate for sparse datasets [23–25].

Here we pair a simple calibration experiment with log-ratio linear models to measure and mitigate the effects of PCR NPM-bias on estimated 16S rRNA sequence composition. Our log-ratio linear models build on the work of Suzuki and Giovannoni and permit modeling of more than two taxa. Our models are also related to those of McLaren et al. [8], yet additionally account for data sparsity and variation due to counting present in typical microbiome studies [23–25]. We couple our models to a calibration framework that allows bias to be estimated directly from microbial community samples without the need to create mock community standards or to develop an isolate library. To validate our approach we design a mock community with known starting composition. We find that even when sequencing many taxa, PCR NPM-bias still follows a consistent log-ratio linear pattern. Additionally, by using 10 random mock communities, we demonstrate that our approach can mitigate bias introduced by PCR. Finally, we apply our approach on complex microbial community samples from an *in vitro* artificial gut model to investigate PCR NPM-bias in real microbial communities.

## Results

### Measuring and modeling PCR bias

We built a model of PCR NPM-bias in two stages: first, we considered a model for PCR amplification of a single template; second we extended this model to PCR NPM-bias in multi-template settings. We denote by *a*_*j*_ the abundance of a transcript *j* ∈ {1, …, *D*} in a pool of DNA prior to PCR amplification. We also denote by *b*_*j*_ the efficiency with which transcript *j* is amplified by PCR, *e.g*., *b*_*j*_ = 2 implies that transcript *j* undergoes perfect doubling at each PCR cycle. Finally, we denote by *w*_*ij*_ the abundance of a transcript *j* in a pool of DNA after *x*_*i*_ cycles of PCR. With this notation we can write the following multiplicative model for PCR of a single transcript:

$$\begin{array}{c} w_{ij}=a_{j}b_{j}^{x_{i}}. \end{array}$$ (1)

Following Suzuki and Giovannoni [7] we extend this model to consider the relative amplification of two transcripts, *j* ∈ {1, 2}, as:

$$\begin{array}{c} \frac{w_{i1}}{w_{i2}}=\frac{a_{1}}{a_{2}}{\left(\frac{b_{1}}{b_{2}}\right)}^{x_{i}}. \end{array}$$

This model simply states that the relative amount of transcript 1 and transcript 2 after *x*_*i*_ cycles of PCR is dependent on the starting ratio of the two transcripts (*a*_1_/*a*_2_) and the ratio of their amplification efficiencies (*b*_1_/*b*_2_). Despite this model’s remarkable simplicity, Suzuki and Giovannoni showed that this model produced a good approximation to observed mid-to-late cycle PCR bias in a two transcript reaction [7]. Importantly, this model is a log-ratio linear model as can be seen by taking the log of both sides:

$$\begin{array}{c} \log\frac{w_{i1}}{w_{i2}}=\log\frac{a_{1}}{a_{2}}+x_{i}\log\frac{b_{1}}{b_{2}}. \end{array}$$ (2)

This observation suggests that, given measurements of transcript relative abundance (*w*_*i*1_/*w*_*i*2_) at different PCR cycle numbers (*x*_*i*_), we can infer the relative abundance of each transcript in the absence of PCR NPM-bias (*a*_1_/*a*_2_), and the relative efficiencies with which the two transcripts are amplified (*b*_1_/*b*_2_)—by simply using linear regression on log-ratio transformed data. That is, in a linear model with *w*_*i*1_/*w*_*i*2_ as the dependent variable and *x*_*i*_ as the independent variable, the relative abundances (*i.e*., proportions) prior to PCR NPM-bias are the intercept and the relative efficiencies are the slope.

To make Eq (2) useful for microbiome studies we must extend it to allow for compositions of many taxa (not just two) and to model count variation and zero values that arises due to sequencing [24, 25]. Fortunately, recent statistical advances allow such models to be inferred efficiently in a straight-forward manner using the R package *fido* even when thousands of microbial taxa are being modeled simultaneously [26, 27]. Through *fido*, multinomial logistic-normal linear models can be fit efficiently, these are a special type of log-ratio linear model that also accounts for the the compositional nature of 16S rRNA high-throughput sequencing data [28, 29] as well as uncertainty due to multivariate counting and zero values [30]. Despite the added complexity, the core concept of the model remains: In a regression of microbial composition versus PCR cycle number, the estimate of a sample’s composition prior to PCR NPM-bias is inferred as the intercept of a log-ratio linear model while the relative efficiency with which each taxon is amplified is represented by the slope.

**Fig 1:**
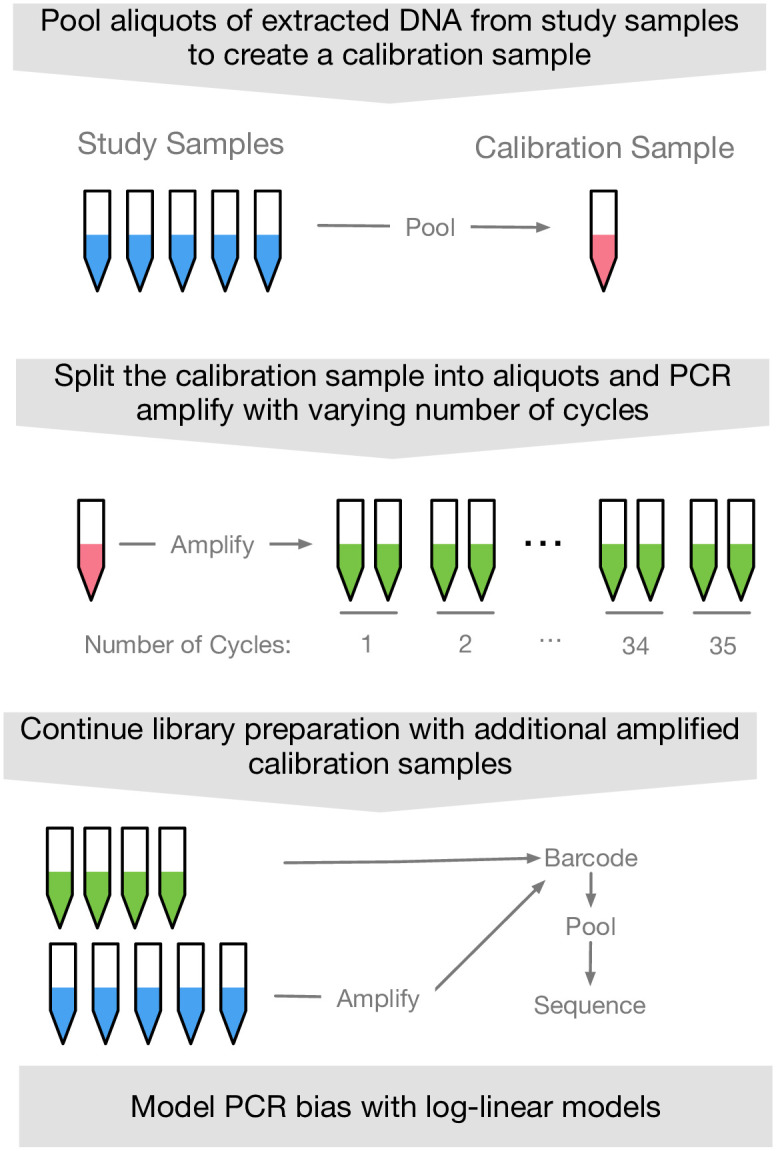
The calibration experiment can be integrated into standard sequencing workflows.

Our approach for measuring and mitigating PCR NPM-bias only requires adding a single calibration experiment to standard sequencing workflows (Fig 1). Our approach can be stated in 4 steps (see Materials and methods for more details). First, prior to PCR, pool aliquots of extracted DNA from each study sample into a single pooled sample (the calibration sample). Pooling the extracted DNA from other samples ensures that each taxon in the study will be represented in the calibration curve. Second, split that sample into aliquots and amplify each aliquot for a predetermined number of PCR cycles (ideally covering as wide a range of PCR cycles as possible while still ensuring that the resultant libraries are detectable by sequencing). Third, treat the resultant calibration samples just like any other sample in the study: barcode, pool, then sequence alongside the study samples. Finally, model those calibration samples with a log-ratio linear model and use the results to mitigate the inferred bias from the remaining samples in the study.

### Mock community analysis

While in practice our approach to measuring and mitigating PCR NPM-bias does not require the creation of mock communities, we developed mock communities in order to validate our approach for use in 16S rRNA studies. We created 11 samples by combining aliquots of DNA extracted from 10 bacterial species in random ratios. One sample was used in our calibration experiment (the calibration sample) while the other 10 were held-out and used to validate our model (the mock communities). Of note, DNA from each isolate had previously gone through single-template PCR (using identical primers) to obtain enough material for mock community creation; as a result, we expect PCR bias from primer mismatch to be absent in our mock communities.

To measure PCR NPM-bias, the calibration sample was split into aliquots and each aliquot underwent a predetermined number of PCR cycles varying from 10 to 35 cycles. To avoid systematic bias from the ordering in which the amplifications were done, the order of PCRs were randomized (Materials and methods). The 10 mock community samples underwent 35 cycles of PCR. The resulting amplified calibration samples and 10 amplified mock community samples were then barcoded, pooled, and sequenced. The resulting table of sequence counts was analyzed using a multinomial logistic-normal linear model from the R package *fido* (Materials and methods) [27]. We also added to our model a random effect term based on the PCR machine to control for batch effects.

The resultant calibration data supported our linear model of PCR NPM-bias. Our linear model explained 95% of the variation in the sequenced calibration curve (mean R2; 95% credible set 94% to 96%; S1 Fig). Moreover we found the calibration curve had a substantial non-zero slope suggesting substantial PCR bias. On average, the relative abundance of each taxon was biased by a factor of 2.6 (95% credible set 2.3 to 2.9) with some taxa such as *C*. *aerofaciens* and *B*. *longum* over-represented by a factor of 4 or more (S2 Fig). In contrast, other taxa such as *E*. *faecalis* were underrepresented by nearly a factor of 4. Similar biases were found when microbial composition was represented in centered log-ratio coordinates (S3 Fig). Together this leads to large compositional shifts due to PCR NPM-bias, for example the relative amount of *B*. *longum* to *E*. *faecalis* is shifted by a factor of approximately 16 due to PCR NPM-bias. Together these results show the substantial effect PCR NPM-bias can have and also support a log-ratio linear model of PCR NPM-bias.

We next sought to evaluate our ability to infer the composition of all 11 of our created samples prior to the introduction of PCR NPM-bias (10 mock communities and 1 calibration sample). Using either Qubit or qPCR to quantify mock community composition, we found that our approach could estimate the wrong direction of PCR bias for certain taxa; for example, saying *B*. *longum* was underrepresented in the calibration sample when it was in fact overrepresented. Yet our results in S1 Fig clearly show the relative abundance of *B*. *longum* increasing with increasing numbers of PCR cycles; that is our calibration data supports the conclusion that *B*. *longum* is over-represented in the calibration sample. To explain this discrepancy, we reasoned that a qPCR-based method for creating a “ground-truth” input concentration to our mock community may itself suffer from PCR bias. Moreover, we expect other biases to be introduced when using genomic DNA concentration as a surrogate for the 16S rRNA gene concentration [31]. Indeed, both these challenges have been previously encountered when specifying the concentration of input species to mock microbial communities [32, 33]. Given these well-known challenges to knowing the true starting concentration of species in our mock community, we therefore performed a follow-up analysis that investigated our ability to reconstruct true relative differences in the abundance of species within mock communities. That is, we generated a vector of correction terms for our mock sample analysis by calculating the difference between our qPCR-based estimates of species concentrations in our mock sample and our inferred abundances of species in our calibration sample. We then applied this correction term to our inferred compositions of 10 separate mock communities whose experimental assembly was performed using the same bacterial concentration values used to compose our calibration sample.

**Fig 2:**
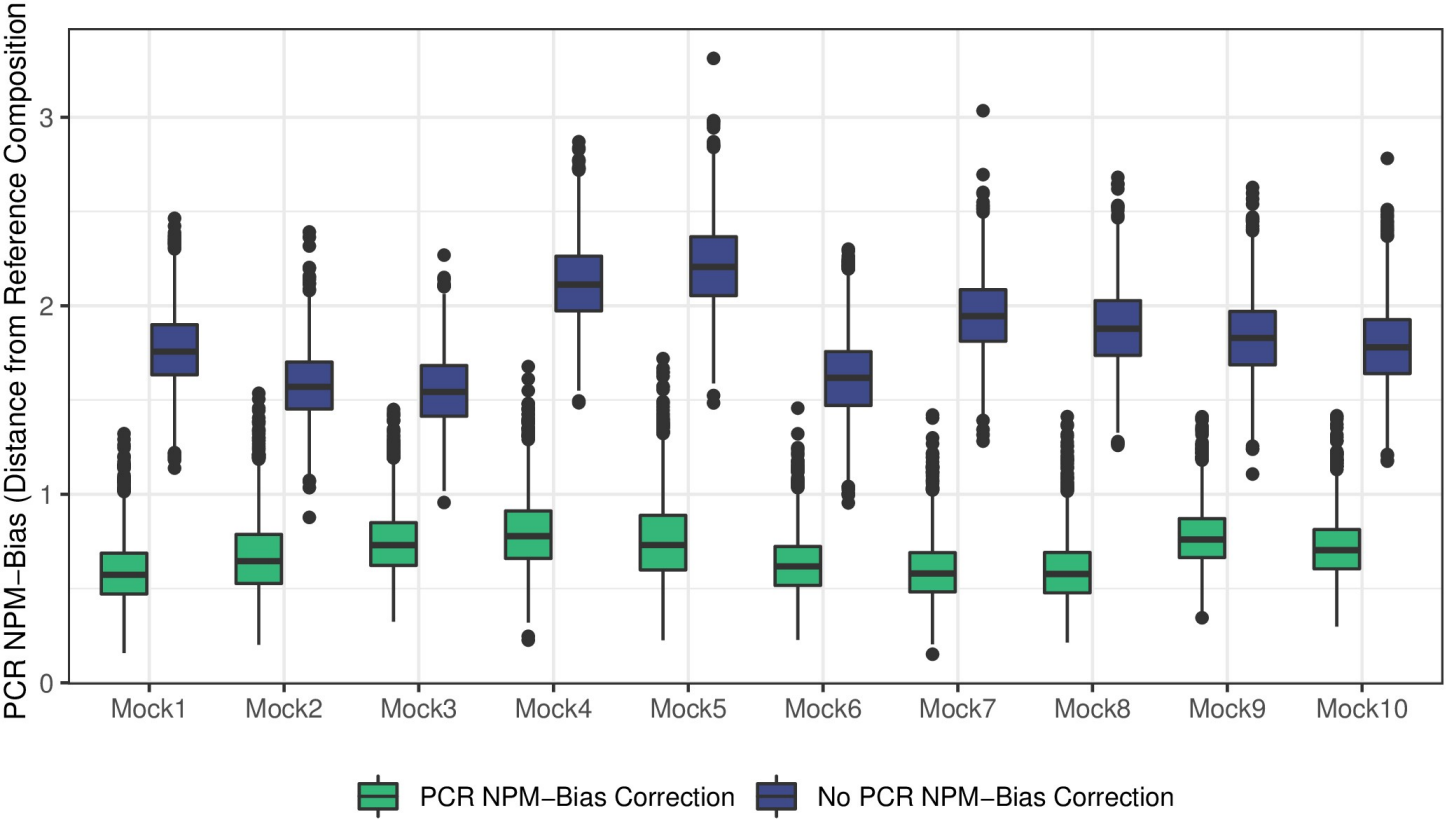
Combining calibration experiments with linear models allows PCR NPM-bias to be mitigated. No bias correction (blue) indicates difference between reference community compositions and raw community composition measured after 35 cycles of PCR. PCR NPM-bias correction (green) indicates the difference (measured by Aitchison distance) to reference community values after PCR bias model applied. Posterior distributions are represented as box plots. PCR NPM-Bias was inferred jointly for four calibration curves each created from a different starting community (Materials and methods). Perfect removal of all PCR NPM-bias in mock community sequenced samples corresponds to a value of 0 on the vertical axis.

In all 10 of the mock communities, our approach produced estimates of sample composition closer to PCR NPM-bias-free composition than the sequenced composition (Fig 2). Moreover, by accounting for PCR NPM-bias, we were able to infer more accurate estimates of alpha diversity for the 10 mock communities. Estimates of Shannon diversity were closer to the true values in 7 of 10 communities, Simpson diversity saw improved estimates in 8 of 10 samples, and Inverse Simpson diversity saw improved estimates in 8 of 10 communities (S4 Fig).

### Human gut microbial community analysis

To characterize and mitigate PCR NPM-bias in human gut microbial communities we repeated the experimental approach used for the mock communities but applied to four different communities derived from human hosts. Rather than using a single calibration experiment on a pooled sample, we performed 4 separate calibration experiments to observe the reproducibility of calibration results starting from different compositions. Each community was cultured *ex vivo* for 1–3 days using an independent artificial gut systems as previously described [30]. The PCR experiments for these human gut microbial communities were performed on multiple PCR machines due to the large number of samples involved. After initial preprocessing, the resulting data represented 68 bacterial genera from 6 bacterial phyla. To fit this data, we modeled each of the four individuals with random intercepts, a fixed effect for cycle number, and random effects for each PCR machine (Materials and methods).

**Fig 3:**
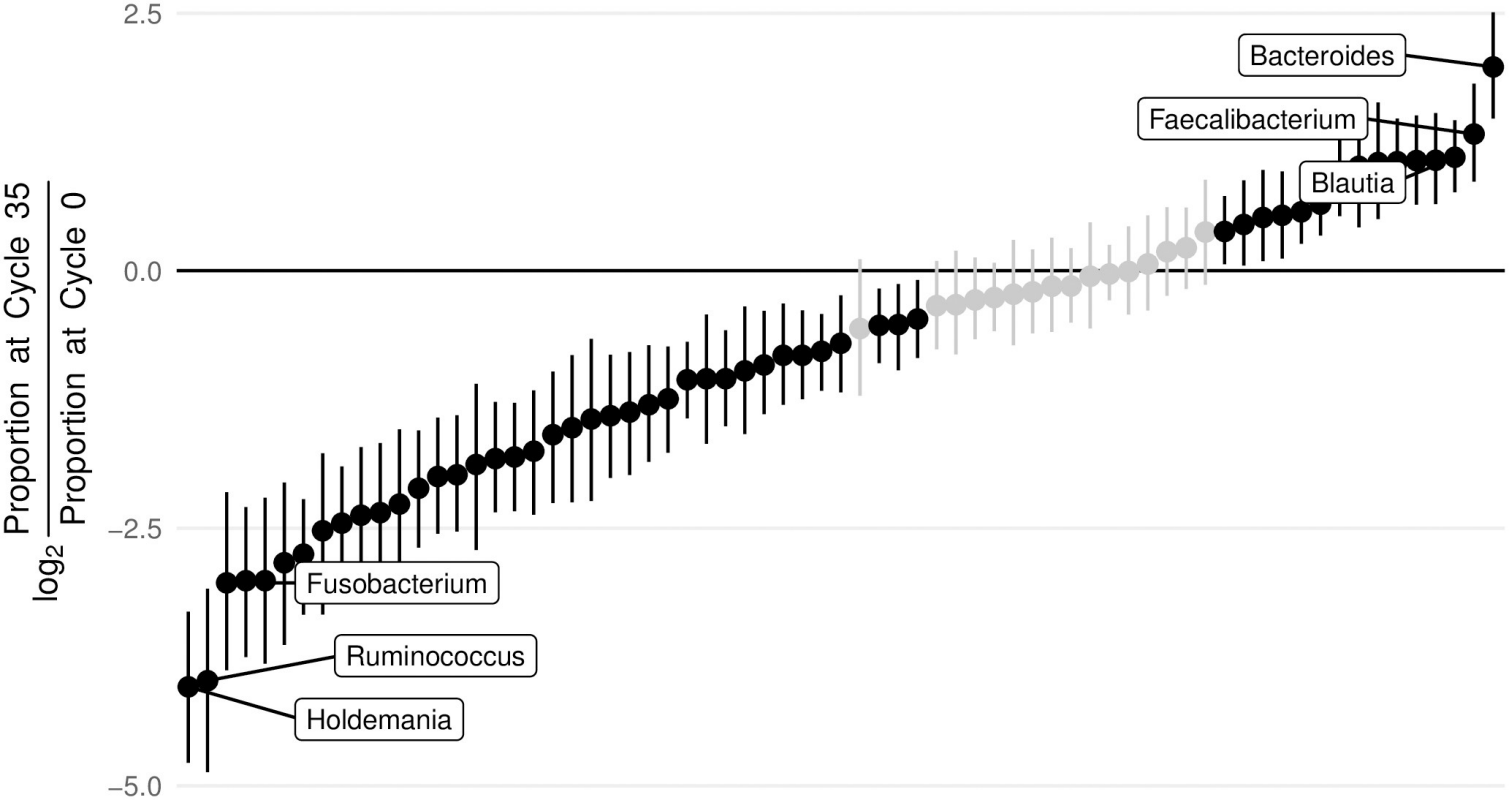
PCR induces substantial bias in human gut microbial communities. To visualize the scale of PCR NPM-bias in human gut microbial communities we calculated NPM-bias induced after 35 cycles of PCR as the log-ratio of the taxon proportion at cycle 35 versus inferred taxon proportions at cycle 0 (unamplified). For example, a value of 2 suggests that a given taxon is over-represented after 35 cycles of PCR by a factor of 4 (2^2^) whereas a value of -2 suggests that a given taxon is underrepresented by a factor of 4. The mean and 95% credible regions for this bias are depicted for each taxon. Those taxa with 95% credible regions not overlapping zero are shown in black. This bias is also presented on the centered log-ratio scale in S5 Fig.

As in our analysis of the mock communities, we found that the calibration data from human gut microbial communities was well fit by a log-ratio linear model. Across 4 separate calibration curves an identical linear relationship between microbial composition and PCR cycle number was able to explain 76% of the variation in the data (95% credible set 73% to 78%). This further supports our conceptual model for PCR NPM-bias in human gut microbial community data. To succinctly visualize the scale of PCR NPM-bias present when amplifying human gut microbial communities, we investigated the total bias introduced into the data after 35 cycles of PCR (Fig 3 and S5 Fig). As in our evaluation of the mock community, we find that 35 cycles of PCR induces a substantial bias in estimated relative abundances (*i.e*., proportions) with approximately 19% of taxa being subject to over a factor of 4 bias. Our results suggest that, for the primers used in this study, the genera *Holdemania*, *Ruminococcus*, and *Fusobacterium* are consistently the most under-represented taxa due to PCR bias while *Bacteroides*, *Faecalibacterium*, and *Blautia* are consistently the most over-represented.

Investigating random effects associated with PCR machine revealed that PCR reactions run on one machine were substantially different than those run on the other machines (S6 Fig). Reviewing the settings on each PCR machine, it was found that the outlying machine had its temperature mis-set during the annealing phase of each PCR cycle (Materials and methods). We therefore excluded data from this machine when estimating bias. More broadly, this finding demonstrates how creating PCR calibration curves can be used to detect and correct for sample processing errors in microbiota surveys.

## Discussion

Here we have presented an approach to characterize and mitigate PCR bias from non-primer mismatch sources (PCR NPM-Bias) in microbiota studies based on calibration experiments and log-ratio linear models. Using both mock and human gut microbial communities, we demonstrated that sequencing data from mid-to-late cycle PCR reactions were well-fit by log-ratio linear models, which lends credence to a conceptual model of PCR NPM-bias as a consistent multiplicative process. Moreover, our model suggests that PCR NPM-bias can alter relative abundance estimates by a factor of 4 or more as well as inducing bias in estimates of alpha diversity. Still, using mock communities we demonstrated that our approach can measure and mitigate this bias. Although we used mock communities to validate our approach, our approach does not require that mock communities be used in everyday practice. We find this appealing as many microbial taxa that may be of interest are difficult to isolate and culture without specialized experimental techniques [22].

It has been hypothesized that analyses that are invariant to compositional perturbation, such as differential changes in log-ratios, may be insensitive to PCR bias whereas others types of analyses may be sensitive [8]. We found that a log-ratio linear model was able to explain 95% of the variation in our mock community calibration curve and 76% of the variation when four independent calibration curves were modeled jointly. These findings support a conceptual model in which PCR NPM-bias is primarily a multiplicative process that is consistent over a wide range of cycles. Consequently, if all samples in a dataset undergo the same number of PCR cycles, then NPM-bias would represent an identical compositional perturbation applied to each sample. Our results therefore suggest that analyses invariant to compositional perturbations will be insensitive to PCR NPM-bias (e.g., log-ratio differences between heath and diseased samples). In contrast, methods that are sensitive to such compositional perturbations, including certain analyses of alpha-diversity or analysis of the most abundant organisms in a community could be highly sensitive to this bias. As expected, our mock community analyses found that Simpsons or Shannon diversity estimates were altered by PCR NPM-bias. Yet other analysis may be more complicated, particularly ones that incorporate knowledge of taxonomic sequence similarity such as Faith’s phylogenetic diversity [34] or UniFrac [35]. In these cases, it remains to be determined whether the incorporation of phylogenetic information would alleviate or aggravate the impact of PCR NPM-bias. Overall, we expect that applying our approach to mitigating this bias could enhance the accuracy of microbiome analyses that are sensitive to compositional perturbations.

Beyond the implications of observing a log-ratio linear relationship between PCR cycle number and microbial composition, we have also provided empirical evidence that our approach can efficiently mitigate PCR NPM-bias. However, this analysis was dependent on our ability to estimate the concentration of 16S rRNA encoding DNA in the DNA extracted from each of the 10 bacterial isolates. As measuring these concentrations by qPCR would share the same bias we were studying, we instead made the assumption that our model was able to correctly predict the composition of the calibration sample at PCR cycle 0 (the composition in the absence of PCR NPM-bias). If PCR NPM-bias in cycles 1 through 9 deviate strongly from the observed log-linear relationship, this assumption could be false and it could appear as though our model was more efficient at mitigating bias than it truly was. Still, the fact that our model was able to explain 95% of the variation in the calibration curve, a total of 69 samples spanning 25 PCR cycles, is consistent with our assumption.

While our empirical results suggest that our approach is capable of mitigating PCR NPM-bias, questions remain regarding how to best apply these methods in practice. Currently, to ensure the calibration curve contains all taxa in a study, we recommend creating the calibration sample by combining extracted DNA from each biological condition. Yet, low abundance taxa may be missed with this pooling method. It is possible that alternative calibration approaches will be superior and provide better bias estimation. For example, future studies could consider using multiple calibration curves, each performed over a small number of PCR cycles and produced from a distinct community chosen to be representative of the overall study. Additionally, our finding that 76% of the variation in 4 independently performed calibration curves could be explained by the same linear relationship suggests that this bias may be highly reproducible. It is therefore possible that calibration curves could be reused between between sequencing runs. We anticipate that further investigation of these questions would prove impactful.

Despite these avenues for further work, we anticipate that our current approach will appeal to researchers seeking to mitigate PCR NPM-bias. Our approach is implemented using the *fido* software package, which provides a flexible framework for building both linear and non-linear models for microbiome sequence count data using Bayesian multinomial logistic-normal models [27]. While inference of this class of models has traditionally been too computationally intensive for general-purpose use, *fido* uses new advances in marginally latent matrix-t processes to perform inference at the scale of thousands of microbial taxa and samples [27]. For example, our analysis of human gut communities in this study took less than 1 minute on a standard laptop computer running on a single core. By using *fido*, it is also possible to incorporate additional covariates into the PCR bias model such as random intercept terms to account for different starting compositions and terms to account for batch effects. A code repository and tutorial are available; links to these are provided in *Data and Code Availability*.

Beyond PCR NPM-bias, other sources of bias remain outstanding challenges for sequencing-based microbial community profiling. Here we have focused on improved estimation of 16S rRNA sequence composition in mixed microbial communities. Yet, bacterial taxa can vary in terms of genomic 16S rRNA copy number, which can lead estimates of bacterial composition from 16S rRNA to differ from the true composition of bacterial cells in a community [36]. Differences in DNA extraction efficiency are another source of bias [8, 32, 37–40]. Even within the PCR process itself, there remain other challenges our method does not directly address. Bias due to PCR primer mismatch [16] is likely not captured by our calibration approach, as mismatches are expected to be prominent in early cycles of PCR that lack sufficient DNA to be sequenced for our calibration curves. Mock community based methods [8] may provide an alternative approach for estimating primer-mismatch bias, but would require assembling and testing a relevant assemblage of microbes for a given sample type. One future line of research could therefore investigate whether PCR NPM-bias is correlated with primer mismatch bias, as shared causal mechanisms would suggest our calibration approach could be adapted to also mitigate primer mismatch bias without the need for mock communities.

## Materials and methods

### PCR bias model

To extend Eq (2) to more than two transcripts we note that any multivariate log-ratio linear model of *D* transcripts can be written in terms of a *D* − 1 × *D* contrast matrix Ψ so that Eq (2) becomes:

$$\begin{array}{ccc} \Psi \log(w_{i}) & =\Psi \log\left(a\right)+\Psi \log\left(b\right)x_{i} & \text{(3)}\\ ↓ & & \\ \eta_{i} & =\alpha +\beta x_{i} & \text{(4)} \end{array}$$

where *η* now represents the relative abundance corresponding to *w*_*i*_ but represented as a vector of log-ratios determined by the contrast matrix Ψ. That is, *η* is defined by the relationship *η*_*i*_ = Ψlog(*w*_*i*_).

Beyond PCR bias, sequence count data may be subject to other sources of technical variation including variation from counting [24] and batch effects. To account for these sources of random variation, we embed Eq (4) in the following probabilistic model

$$\begin{array}{c} Y_{i}∼\text{Multinomial}(\pi_{i}) \end{array}$$ (5)

$$\begin{array}{c} \pi_{i}=\phi^{-1}\left(\eta_{i}\right) \end{array}$$ (6)

$$\begin{array}{c} \eta_{i}∼N(\Lambda X_{i},\Sigma ) \end{array}$$ (7)

where *Y*_*i*_ denotes the sequence counts from a sample *i* ∈ {1, …, *D*}, Λ*X*_*i*_ denotes a generalization of *α* + *βx*_*i*_ to a larger class of linear models (*e.g*., allowing other covariates such as batch number to be modeled in addition to PCR cycle number), and *ϕ*^−1^(*η*_*i*_) denotes the inverse transformation of *η*_*i*_ = Ψlog(*π*_*i*_) which is given by $\pi_{i}=C\left[\exp\left({\left(\Psi^{†}\right)}^{T}\eta_{i}\right)\right]$, where (Ψ^†^)^*T*^ is defined by the relation (Ψ^†^)^*T*^Ψ = *I*_*D*−1_, and where C denotes the closure operation defined as

$$\begin{array}{c} C\left[m_{1},\cdots ,m_{D}\right]=(\frac{m_{1}}{\sum_{j=1}^{D}m_{j}},\cdots ,\frac{m_{D}}{\sum_{j=1}^{D}m_{j}}). \end{array}$$ (6)

Eqs (5)–(7) denote a multinomial logistic-normal linear model. In this work we fit a Bayesian formulation of the above model using matrix-normal and inverse Wishart priors

$$\begin{array}{c} \Lambda ∼N(\Theta ,\Sigma ,\Gamma ) \end{array}$$ (8)

$$\begin{array}{c} \Sigma ∼IW(\Xi ,\upsilon ) \end{array}$$ (9)

which is available as the function *pibble* in the *fido* R package [27] which performs inference using a marginal Laplace approximation to the latent matrix-t representation of this model [26]. Together, Eqs (5)–(9) form a generative model for PCR bias in sequence count data motivated by the log-ratio linear model of PCR bias given in Eq (2).

### Sample acquisition

Fecal samples were collected from four human subjects under a protocol approved by the Duke Health Institutional Review Board (Duke Health IRB Pro00049498). Subjects provided fecal samples at no risk to themselves, had no acute enteric illness, and had not taken antibiotics in the past month.

### Mock community data collection

Mock communities were created using ten bacterial isolates selected to be distinguishable by 16S rRNA sequencing. The following reagents were obtained through BEI Resources, NIAID, NIH as part of the Human Microbiome Project: Hungatella hathewayi, Strain WAL-18680, HM-308; Streptococcus gallolyticus subsp. gallolyticus, Strain TX20005, HM-272; and Lactobacillus oris, Strain F0423, HM-560. The following reagent was obtained through DSMZ German Collection of Microorganisms and Cell Culture GmbH: Roseburia intestinalis, Strain L1–82, DSM No. 14610, Type strain. The remaining seven isolates were isolated and cultured from human fecal samples: Bacillus subtilis, Bifidobacterium longum, Collinsella aerofaciens, Clostridium innocuum, Enterobacter faecalis, and Lactobacillus ruminis.

DNA from individual cultures were extracted using Qiagen UltraClean kits. The concentration of total DNA extracted and amplified from each isolate was quantified using Quant-iT dsDNA Assay Kit (Thermo Fisher Scientific). Eleven mock communities were created based on the Quant-iT based concentrations. One mock community (the calibration sample) was created by combining equal amounts of DNA from each of the 10 isolates. The other 10 mock communities were created by sampling uniformly from a 10 dimensional simplex with the constraint that the maximum fold change between any two isolate concentrations was less than or equal to 10. This later constraint was added to ensure the resultant random community compositions fell within the dynamic range of standard laboratory pipettes. As Quant-iT quantifies total DNA, not just 16S rRNA, qPCR was also used to estimate the resulting mock community composition based on amplifying 16S rRNA. qPCR was performed as follows: the V4 region of the 16S rRNA gene was amplified (F515/R806) [1]; all reactions began with a denaturing step of 94C for 3 minutes, followed by 35 amplification cycles—one amplification cycle consists of: 94C for 45 seconds, 50C for 1 minute, 72C for 1.5 minutes—and finished with 10 minutes of 72C. A calibration curve as described in Fig 1 was created using the calibration sample. PCR was performed using the same primers as qPCR. Primers were barcoded. PCR steps were adapted from Caporaso et al. to permit a variable number of PCR cycles: all reactions began with a denaturing step of 94C for 3 minutes, followed by a variable number of amplification cycles, and finished with 10 minutes of 72C. One amplification cycle consists of: 94C for 45 seconds, 50C for 1 minute, 72C for 1.5 minutes. Samples were collected from all amplification cycles between 10 and 35. To avoid systematic bias, the order in which the PCRs were done was randomized by using the function ‘sample’ in the R programming language applied to the PCR cycle numbers included in the calibration curves. The other 10 mock communities underwent 35 cycles of PCR using identical protocols as used for the calibration sample. Samples created as part of the calibration curve were pooled along with samples from the 10 mock communities. 16S rRNA amplicon sequencing was performed using an Illumina MiniSeq with paired-end 150 bp reads.

### Human gut microbial community data collection

To characterize PCR bias for human gut microbial communities we analyzed samples from an artificial gut system. Four fecal samples from four separate donors were used to inoculate artificial gut vessels as previously reported [30]. To obtain enough starting material, fecal samples from each donor were obtained by pooling fecal material from the inoculum, Day 1, Day 2, and Day 3 of each artificial gut vessel. Bacterial DNA was extracted using Qiagen DNeasy PowerSoil Kit. The bacterial DNA concentration of the samples was quantified using a Quant-iT dsDNA Assay Kit (Thermo Fisher Scientific). As in the mock community, the V4 region of the 16S rRNA gene was barcoded and amplified. Four separate calibration curves were created from the four fecal samples. PCR was performed using the same parameters as for the mock community except PCR amplifications were split between 5 machines. Samples were collected from all amplification cycles between 20 and 35. 16S rRNA amplicon sequencing was done by an Illumina MiniSeq with paired-end 150 bp reads. After initial data analysis it was found that PCR machine 3 was miscalibrated and the middle amplification step was set to 58C rather than 50C. As a result, samples from machine 3 were excluded from subsequent analyses.

### Data preprocessing

Sequencing data was processed and denoised using DADA2 [41] following a previously published analysis pipeline [30]. For both the mock and human gut microbial community data, only samples with more than 1000 reads were retained for analysis. This retained 99.9% of sequence variant counts from the mock and 99.8% of sequence variant counts from the human gut microbial communities respectively. The mock community data was analyzed at the sequence-variant level. Sequence variants were mapped to isolates based on minimum Levenshtein distance [42]. The human gut microbial community data was analyzed at the genus level and genera that were not seen in at least 30% of samples with at least 3 counts were amalgamated together into a category called “other” for analysis. We chose to analyze these data at the genus level, rather than the sequence variant level, so that, for simplicity, we could reference taxa using taxonomic designations which are frequently not present at the sequence variant level. The *fido* software package scales to thousands of taxa and as such, this data could alternatively have been analyzed at the sequence variant level. Notably, no pseudo-counts were added to the data prior to analysis as the Bayesian multinomial-logistic normal linear model in Eqs (5)–(9) models zeros directly [25].

### Analysis of mock community data

To model the mock community data we took *X*_*i*_ (the covariate vector assigned to sample *i* to be $X_{i}={\left[I_{{\text{Mock}}_{0}},\ldots ,I_{{\text{Mock}}_{10}},x_{i},I_{{\text{PCR}}_{2}},I_{{\text{PCR}}_{3}},I_{{\text{PCR}}_{4}}\right]}^{T}$ where 1 represents a constant intercept, *x*_*i*_ denotes the number of PCR cycles that sample *i* went through, and $I_{{\text{PCR}}_{2}}$ is a binary variable denoting whether that sample was amplified on the second (of four) PCR machines, and $I_{{\text{Mock}}_{i}}$ is a binary variable denoting whether the sample is from the *i*-th mock community (with *i* = 0 being the calibration sample). This specification for *X*_*i*_ implies that Λ can be interpreted as

$$\begin{array}{c} \Lambda =[\begin{array}{ccccccc} \alpha_{1}^{\left(0\right)} & \cdots & \alpha_{1}^{\left(10\right)} & \beta_{1} & \gamma_{1}^{\left(2\right)} & \gamma_{1}^{\left(3\right)} & \gamma_{1}^{\left(4\right)}\\ \vdots & \vdots & \vdots & \vdots & \vdots & \vdots & \vdots \\ \alpha_{D-1}^{\left(0\right)} & \cdots & \alpha_{D-1}^{\left(10\right)} & \beta_{D-1} & \gamma_{D-1}^{\left(2\right)} & \gamma_{D-1}^{\left(3\right)} & \gamma_{D-1}^{\left(4\right)} \end{array}] \end{array}$$

where $\alpha_{\ell }^{\left(i\right)}$ represents the *ℓ*-th log-ratio of the *i*-th mock community at cycle 0, *β*_*ℓ*_ is per-cycle bias of the *ℓ*-th log-ratio, and *γ*_*ℓ*_ is a variable we introduce to model potential batch effects on the *ℓ*-th log-ratio introduced by using different PCR machines.

We choose to use a weak Bayesian prior for PCR bias encoded as Γ = *σ*^2^ *I*_15_ where *I*_15_ represents a 15 × 15 identity matrix (15 being the number of covariates in *X*) and Θ = 0_(*D*−1)×15_. A value of *σ*^2^ = 10 was chosen by maximum marginal likelihood when the above model was applied to the calibration samples. Additionally, our prior reflected our weak belief that the covariance between the absolute abundance of taxa was independent on the log-scale (Ξ = Ψ*I*Ψ^*T*^ and *υ* = *D* + 2). The multinomial logistic-normal linear model was fit in additive log-ratio coordinates as is default in *fido* and the resulting posterior samples were then transformed into the centered log-ratio coordinate system for figure generation. This transformation was performed using the function *to_clr* provided by the *fido* software package.

### Analysis of human gut microbial community data

To model the human gut microbial community data we took *X*_*i*_ to be $X_{i}={\left[I_{P_{1}},\ldots ,I_{P_{4}},x_{i},I_{{\text{PCR}}_{2}},\ldots ,I_{{\text{PCR}}_{5}}\right]}^{T}$ where $I_{P_{1}}$ is a binary variable denoting if the *i*-th sample was from person 1, *x*_*i*_ denotes the PCR cycle number as in the mock community, and $I_{{\text{PCR}}_{2}}$ is a binary variable denoting if the *i*-th sample was amplified on PCR machine number 2.

Based on our analysis of the mock community data we updated our prior to better reflect our updated beliefs. We choose Γ = diag(4, 4, 4, 4, 1, 1, 1, 1, 1) reflecting our updated prior belief regarding the relative scale of the community intercept and other covariates. In this way we used a form of Bayesian sequential learning to update our prior beliefs for the human gut microbial community data based on the posterior estimates from the mock community analysis. As before we took Θ to be a matrix of zeros. Ξ and *υ* were chosen as in the mock community analysis. The multinomial model was fit and posteriors transformed as in the analysis of the mock community data.

## Supporting information

S1 Fig Calibration curve for mock community data. The marginal regression line for the relation between PCR cycle number and microbial composition (mean and 95% credible set). While systematic bias due to the use of multiple PCR machines (shown as different shaped points) was modeled as a random effect in the regression, for simplicity, their effects are not shown in the marginal regression line. Multivariate R2 statistics were calculated for each posterior sample and had a mean of 95% and a 95% credible set of 94% to 96%.
(TIF)

S2 Fig Bias visualized for mock community data, proportions. To visualize the scale of PCR bias in the calibration sample we calculated bias induced after 35 cycles of PCR as the log-ratio of the taxon proportion at cycle 35 versus inferred taxon proportions at cycle 0 (unamplified). For example, a value of 2 suggests that a given taxon is over-represented after 35 cycles of PCR by a factor of 4 (2^2^) whereas a value of -2 suggests that a given taxon is underrepresented by a factor of 4. The mean and 95% credible regions for this bias is depicted for each taxon. Those taxa with 95% credible regions not overlapping zero are shown in black. A similar figure but made using centered log-ratio coordinates is given in S3 Fig.
(TIF)

S3 Fig Bias visualized for mock community data, centered log-ratios (CLR). To visualize the scale of PCR bias in the calibration sample we calculated bias induced after 35 cycles of PCR as the difference of the taxon CLR coordinate at cycle 35 versus inferred taxon CLR coordinate at cycle 0 (unamplified). The mean and 95% credible regions for this bias is depicted for each taxon. Those taxa with 95% credible regions not overlapping zero are shown in black.
(TIF)

S4 Fig Accounting for PCR NPM-bias produces more accurate estimates of alpha diversity. For each of the 10 mock communities, each of the three different alpha diversity measures (Inverse Simpsons, Shannon’s and Simpsons) were calculated for the true compositions. In addition, for each posterior sample from the model, the same three diversity measures were calculated for the composition after 35 PCR cycles (No PCR NPM-Bias Correction) and the composition inferred unamplified composition (PCR NPM-Bias Correction). The closeness of the true alpha diversity value to the two posterior distributions (PCR NPM-Bias Correction and No PCR NPM-Bias Correction) was evaluated as the empirical cumulative distribution function for each posterior distribution evaluated at the true value and centered about zero. That is, a value of zero is optimal performance and indicates that the true value fell right in the middle (at the median) of the posterior distribution; in contrast, a value of .36 indicates that the posterior distribution has an extra 36% of its mass below the true value whereas a value of -.29 indicates that the posterior distribution had an extra 29% of its mass above the true value. Therefore values closer to zero in absolute value are considered to be better. This statistic is used to summarize, in a single statistic, both the accuracy of the posterior mean as well as the uncertainty about that mean.
(TIF)

S5 Fig PCR NPM-bias visualized for human gut microbial community data, centered log-ratios (CLR). To visualize the scale of PCR bias in the calibration sample we calculated bias induced after 35 cycles of PCR as the difference of the taxon CLR coordinate at cycle 35 versus inferred taxon CLR coordinate at cycle 0 (unamplified). The mean and 95% credible regions for this bias is depicted for each taxon. Those taxa with 95% credible regions not overlapping zero are shown in black.
(TIF)

S6 Fig Posterior euclidean norm of random intercept vector associated with each PCR machine from human gut microbial community data analysis. This norm is shown as a kernel density estimate over 2000 posterior samples for each PCR machine.
(TIF)
