# Supplementary figures and images for: Measuring and mitigating PCR bias in microbiota datasets
Source: PLoS Comput Biol. 2021 Jul 6;17(7):e1009113. doi: 10.1371/journal.pcbi.1009113 (PMC8284789; doi:10.1371/journal.pcbi.1009113)

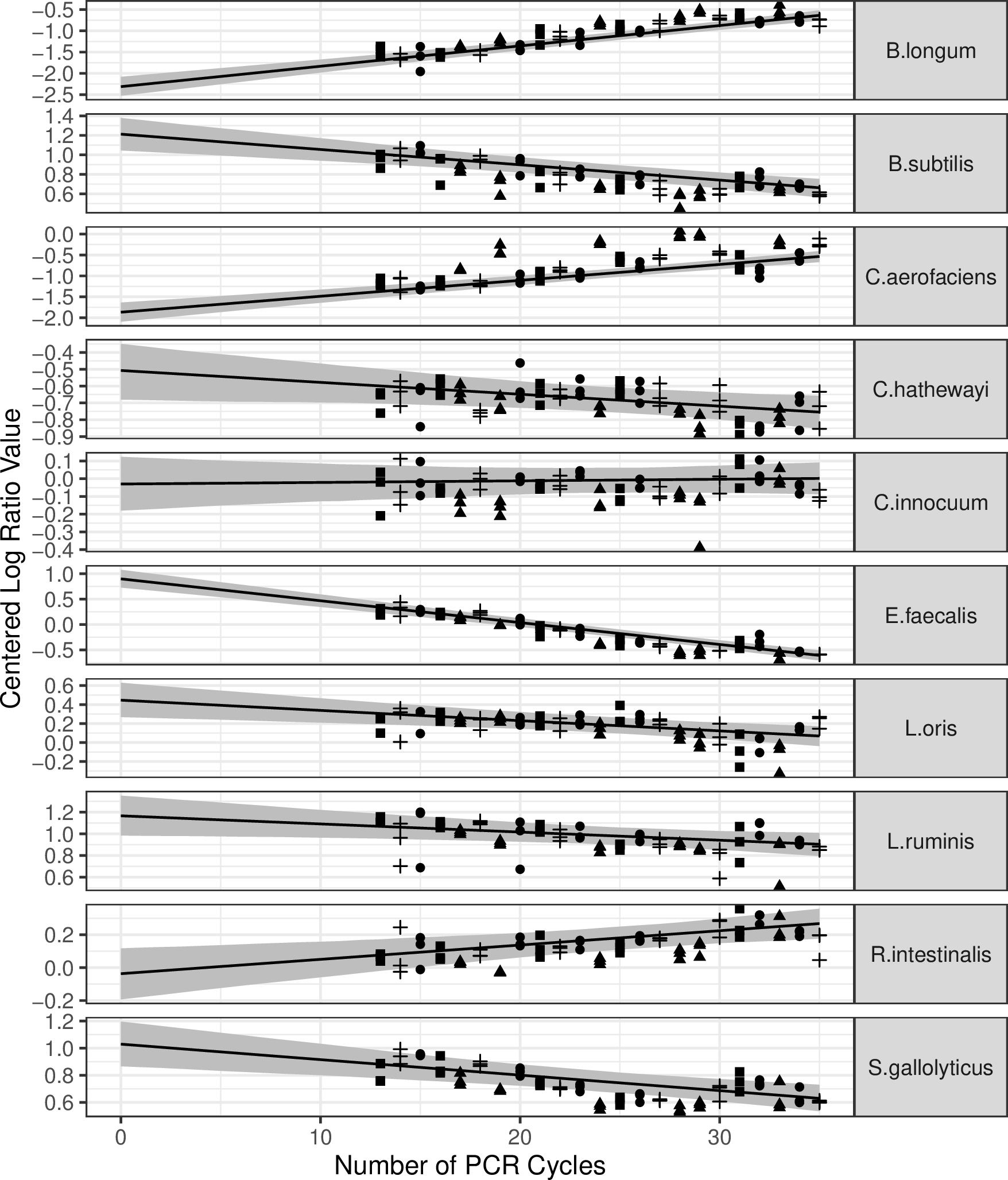

Supplement: S1 Fig — The marginal regression line for the relation between PCR cycle number and microbial composition (mean and 95% credible set). While systematic bias due to the use of multiple PCR machines (shown as different shaped points) was modeled as a random effect in the regression, for simplicity, their effects are not shown in the marginal regression line. Multivariate R2 statistics were calculated for each posterior sample and had a mean of 95% and a 95% credible set of 94% to 96%. (TIF) [file pcbi.1009113.s001.tif]

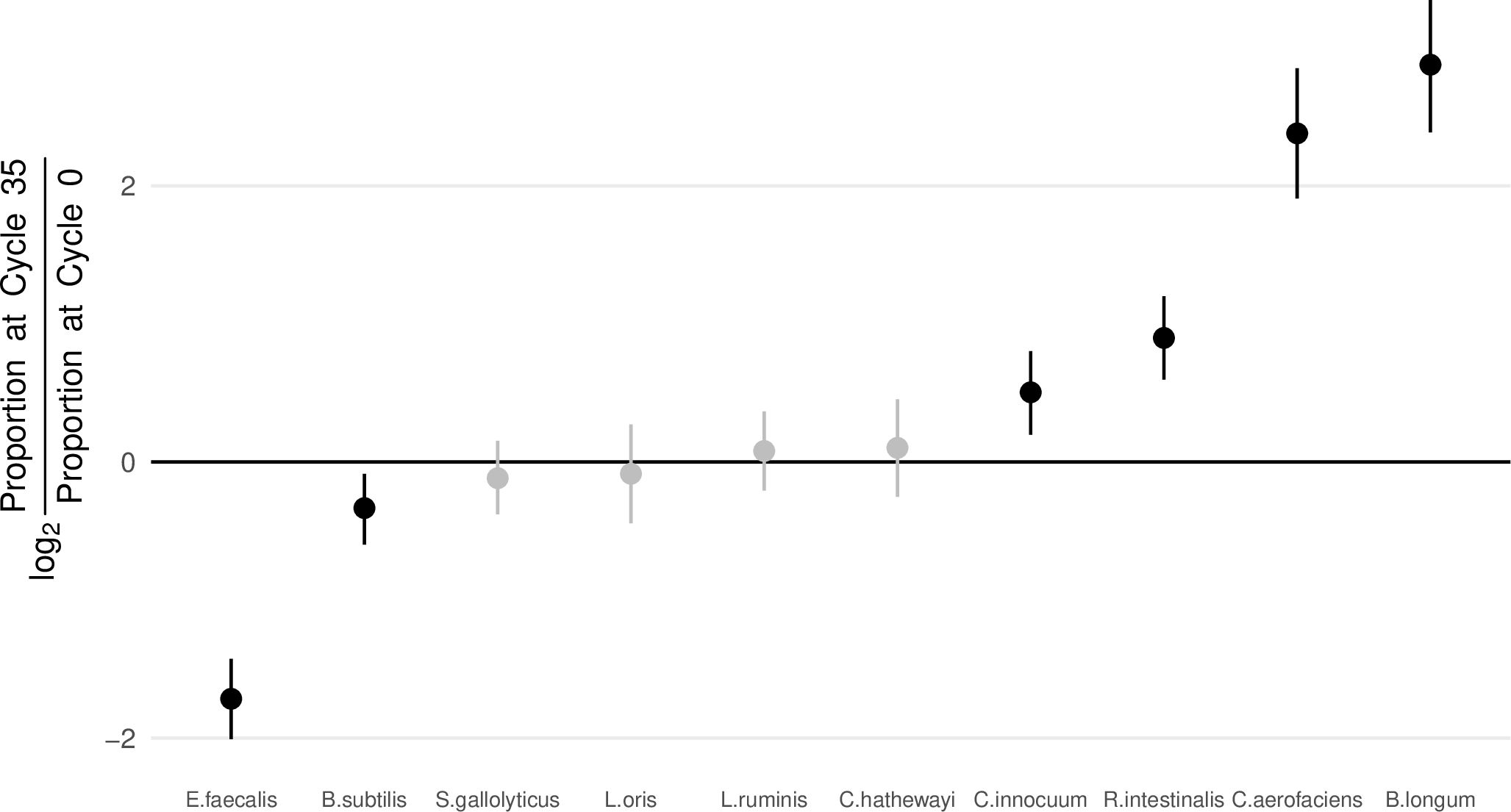

Supplement: S2 Fig — To visualize the scale of PCR bias in the calibration sample we calculated bias induced after 35 cycles of PCR as the log-ratio of the taxon proportion at cycle 35 versus inferred taxon proportions at cycle 0 (unamplified). For example, a value of 2 suggests that a given taxon is over-represented after 35 cycles of PCR by a factor of 4 (22) whereas a value of -2 suggests that a given taxon is underrepresented by a factor of 4. The mean and 95% credible regions for this bias is depicted for each taxon. Those taxa with 95% credible regions not overlapping zero are shown in black. A similar figure but made using centered log-ratio coordinates is given in S3 Fig. (TIF) [file pcbi.1009113.s002.tif]

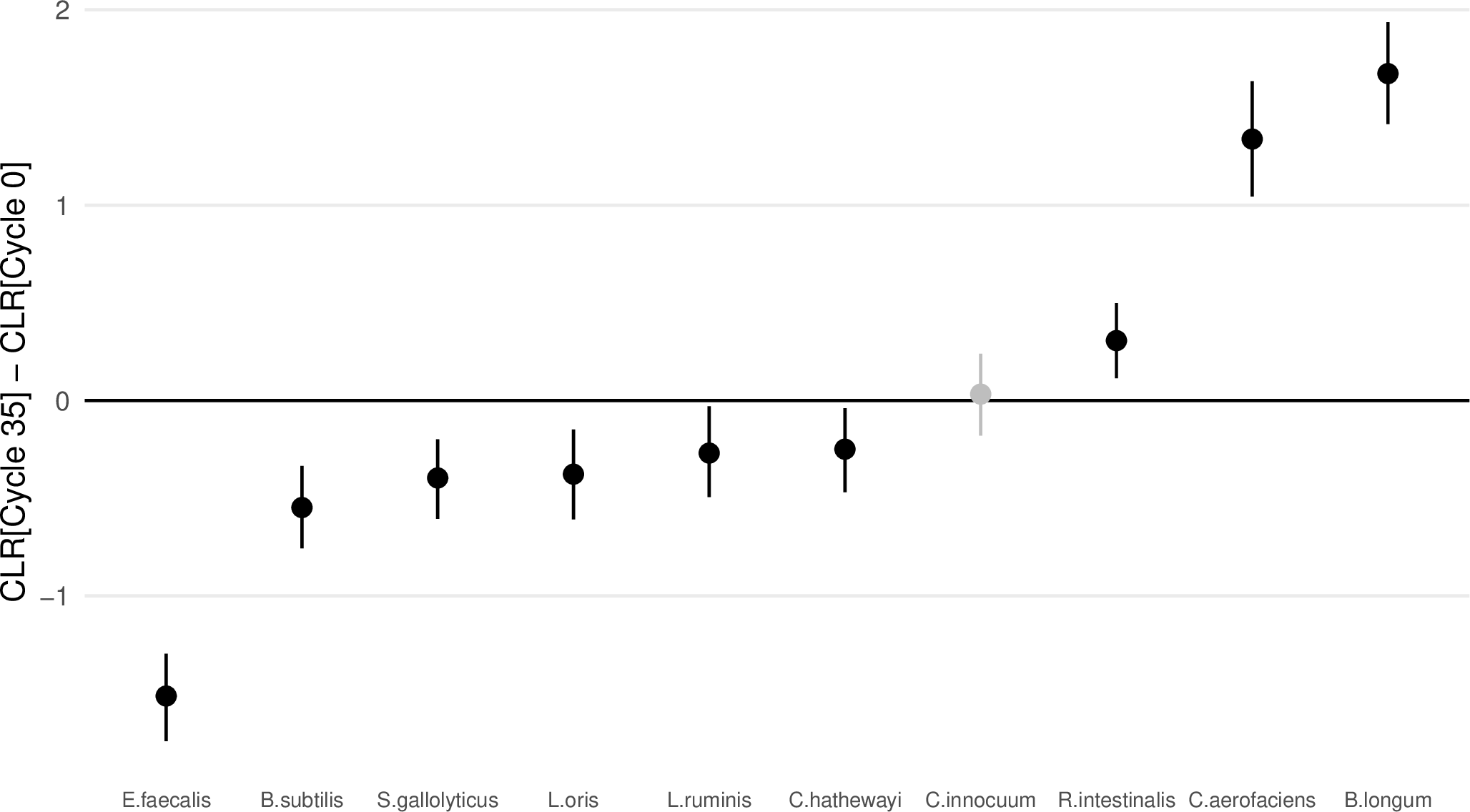

Supplement: S3 Fig — To visualize the scale of PCR bias in the calibration sample we calculated bias induced after 35 cycles of PCR as the difference of the taxon CLR coordinate at cycle 35 versus inferred taxon CLR coordinate at cycle 0 (unamplified). The mean and 95% credible regions for this bias is depicted for each taxon. Those taxa with 95% credible regions not overlapping zero are shown in black. (TIF) [file pcbi.1009113.s003.tif]

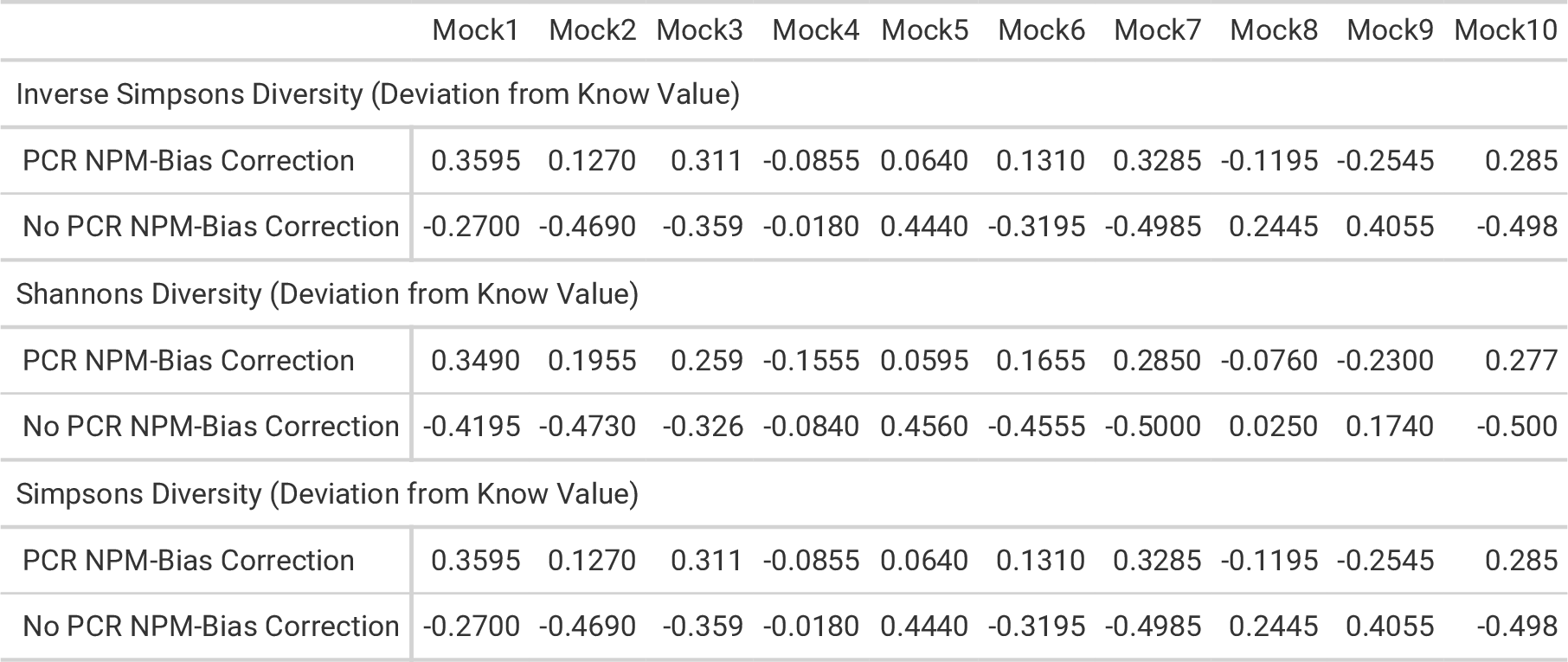

Supplement: S4 Fig — For each of the 10 mock communities, each of the three different alpha diversity measures (Inverse Simpsons, Shannon’s and Simpsons) were calculated for the true compositions. In addition, for each posterior sample from the model, the same three diversity measures were calculated for the composition after 35 PCR cycles (No PCR NPM-Bias Correction) and the composition inferred unamplified composition (PCR NPM-Bias Correction). The closeness of the true alpha diversity value to the two posterior distributions (PCR NPM-Bias Correction and No PCR NPM-Bias Correction) was evaluated as the empirical cumulative distribution function for each posterior distribution evaluated at the true value and centered about zero. That is, a value of zero is optimal performance and indicates that the true value fell right in the middle (at the median) of the posterior distribution; in contrast, a value of .36 indicates that the posterior distribution has an extra 36% of its mass below the true value whereas a value of -.29 indicates that the posterior distribution had an extra 29% of its mass above the true value. Therefore values closer to zero in absolute value are considered to be better. This statistic is used to summarize, in a single statistic, both the accuracy of the posterior mean as well as the uncertainty about that mean. (TIF) [file pcbi.1009113.s004.tif]

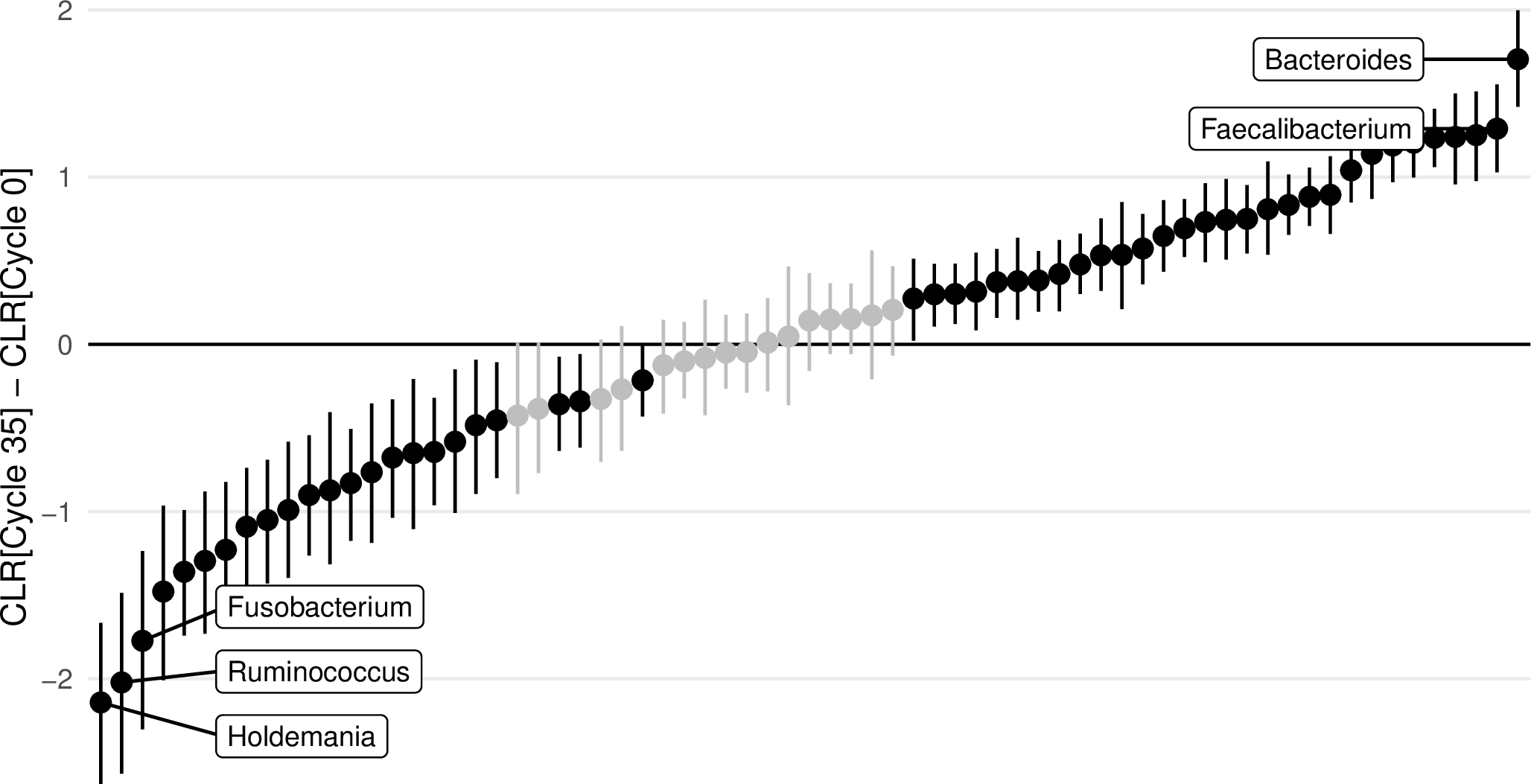

Supplement: S5 Fig — To visualize the scale of PCR bias in the calibration sample we calculated bias induced after 35 cycles of PCR as the difference of the taxon CLR coordinate at cycle 35 versus inferred taxon CLR coordinate at cycle 0 (unamplified). The mean and 95% credible regions for this bias is depicted for each taxon. Those taxa with 95% credible regions not overlapping zero are shown in black. (TIF) [file pcbi.1009113.s005.tif]

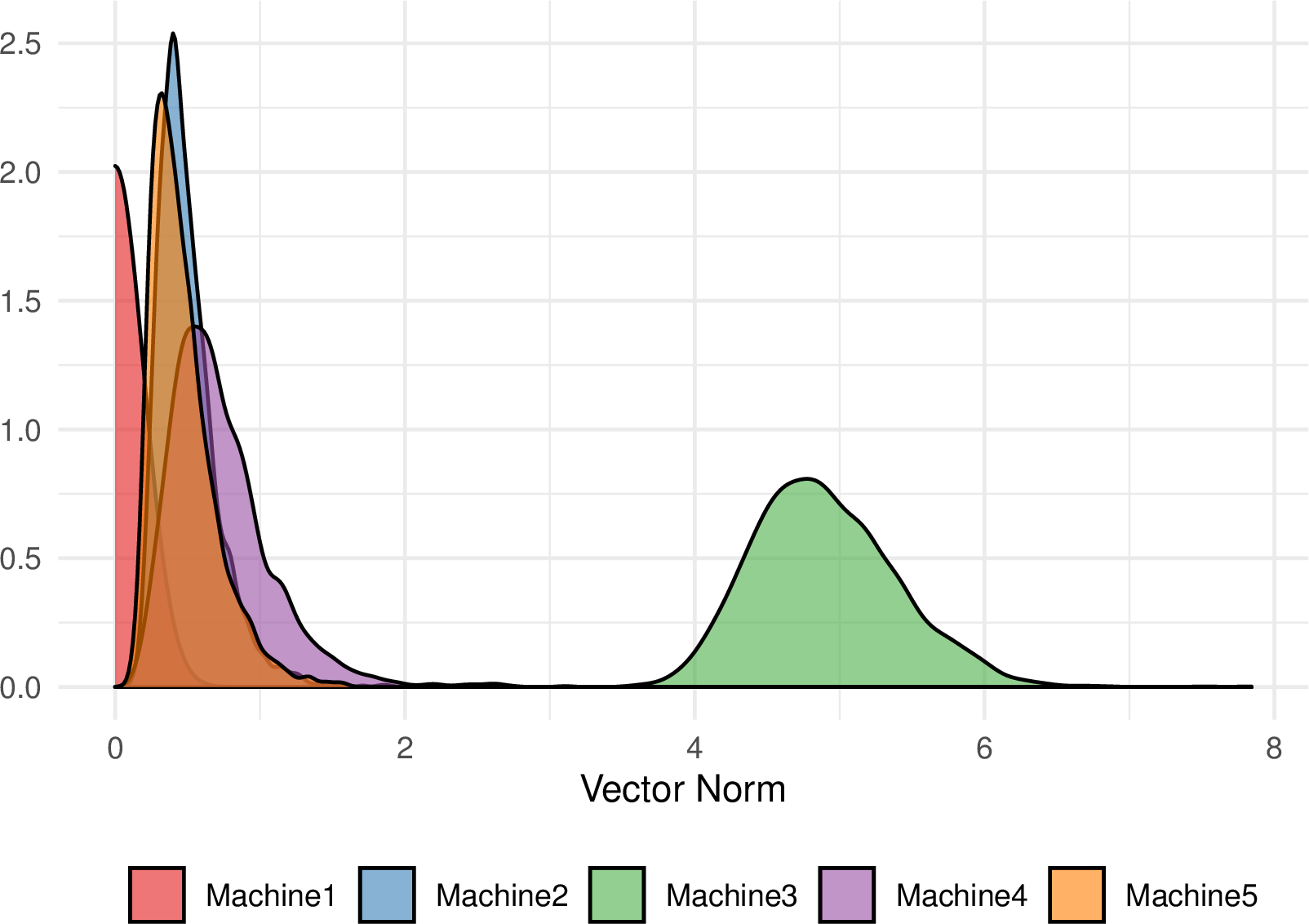

Supplement: S6 Fig — This norm is shown as a kernel density estimate over 2000 posterior samples for each PCR machine. (TIF) [file pcbi.1009113.s006.tif]
